# Preventing Alcohol and Tobacco Use Through Life Skills Training: Theory, Methods, and Empirical Findings

**Published:** 2000

**Authors:** Gilbert J. Botvin, Lori Wolfgang Kantor

**Affiliations:** Gilbert J. Botvin, Ph.D., is a professor and director of the Institute for Prevention Research at Cornell University Medical College in New York City and Lori Wolfgang Kantor, M.A., is a science editor of Alcohol Research & Health

**Keywords:** school-based prevention, life skills, self-management skills, skills building, adolescent, attitude toward AODs (alcohol or other drugs), program evaluation, prevention outcome, prevention approach, social learning theory of AODU (AOD use, abuse, and dependence)

## Abstract

Rates of drinking and smoking increase among high school students as they age. Therefore, prevention programs that target youth either before or during junior high school may help prevent alcohol, tobacco, and other drug (ATOD) use during high school. Life skills training (LST) is a school-based approach designed to prevent ATOD use among youth by influencing their knowledge and attitudes about ATODs, by teaching skills for resisting social pressures to use ATODs, and by helping students develop personal self-management and social skills. Researchers have studied this program’s effectiveness in preventing use of various substances among varied populations.

Both alcohol and tobacco use are common among teenagers in the United States. According to results of a 1999 survey (Johnston et al. 2000), 24 percent of 8th graders, 40 percent of 10th graders, and 51 percent of 12th graders reported drinking alcohol within the past month. In addition, 15 percent of 8th graders, 26 percent of 10th graders, and 31 percent of 12th graders reported engaging in binge drinking (i.e., having five or more drinks in a row) at least once during the 2 weeks before the survey was conducted. Compared with drinking, smoking is less prevalent among high school students. For example, according to the study’s findings, 18 percent of 8th graders, 26 percent of 10th graders, and 35 percent of 12th graders reported smoking cigarettes during the month before the survey, and only 8 percent of 8th graders, 16 percent of 10th graders, and 23 percent of 12th graders reported smoking cigarettes on a daily basis (Johnston et al. 2000).

These survey results indicate that the prevalence of both alcohol use and tobacco use increases with age. Therefore, prevention programs should target youth before or during junior high school. This article describes an approach to substance abuse prevention called life skills training (LST) and summarizes nearly two decades of research on the effectiveness of the LST program in varied populations.

## The Life Skills Training Program

The LST program was designed as a school-based intervention to target a specific set of risk factors for alcohol, tobacco, and other drug (ATOD) use. Thus, LST is a primary prevention program. The ultimate goal of primary prevention programs, which target younger populations, such as junior high school students, is to reduce the prevalence of ATOD use and abuse among youth as they age.

### Background and Rationale

Etiology research has identified several important risk factors for adolescent ATOD use as well as several protective factors that are important in countering the effects of these risks (Hawkins et al. 1992). As shown in the figure, factors contributing to the initiation of ATOD use can be clustered into several broad categories, including the following:

- Sociocultural (e.g., demographic factors, acculturation, and ethnic identity)
- Family factors (e.g., family management practices, discipline, monitoring, and parental ATOD use)
- Social environment factors (e.g., availability of ATODs, school bonding, media influences, and peer influences)
- Cognitive expectancies (e.g., attitudes and beliefs about ATOD use in general and ATOD use among peers [i.e., normative expectations])
- Personal and social competence skills (e.g., decision-making, anxiety management skills, communication skills, and assertiveness)
- Psychological factors (e.g., self-efficacy, self-esteem, and psychological well-being).

These etiologic factors have been incorporated into a comprehensive model of ATOD use initiation. In this model (see figure), ATOD use is conceptualized as the result of a dynamic interaction of environmental and individual factors in which peers, parents, and other social influences interact with individual psychosocial vulnerabilities to promote ATOD use. For example, some people may be influenced to use ATODs by media presentations that normalize or glamorize ATOD use, whereas other people may be influenced by family members or friends who use ATODs or hold attitudes and beliefs supportive of their use. These social influences are likely to have the strongest effect on people with pro-ATOD normative expectations, poor social and personal competence skills, and poor drug-resistance skills. Social influences promoting ATOD use are also likely to have a greater effect on people with certain psychological vulnerabilities, such as social anxiety, low self-esteem, low self-efficacy, and psychological distress. The more risk factors that a person has, the greater is the likelihood that the person will use or abuse ATODs.

This theoretical framework contains key elements from several prominent etiological theories of ATOD use, including social learning theory (Bandura 1977); problem behavior theory (Jessor and Jessor 1977); and self-derogation (Kaplan 1980), persuasive communications (McGuire 1968), and peer cluster theories (Oetting and Beauvais 1987).

In addition to organizing key factors associated with initiation of ATOD use, the model in the figure helps conceptualize potential points of intervention for ATOD abuse prevention. For example, a prevention program that improves personal and social competence skills may have a beneficial effect on various psychological factors associated with reduced ATOD risk. This type of model, however, also has some inherent limitations. The model does not capture the dynamic and recursive nature of the mechanisms by which risk and protective factors act, nor does it contain an exhaustive list of etiological factors.

## Program Overview

The LST program was designed to influence factors at the individual level, as shown in the figure below, through the development of three components: (1) to influence ATOD-related knowledge, attitudes, and norms; (2) to teach skills for resisting social influences to use ATODs; and (3) to promote the development of personal self-management and social skills. Following is a brief description of each of these components:

- *ATOD-related information and skills.* This component is designed to influence ATOD-related knowledge and attitudes, normative expectations, and skills for resisting media and peer influences to use ATODs. The material examines (1) both the short- and long-term consequences of ATOD use, (2) the actual levels of ATOD use among adults and adolescents (to correct normative expectations about ATOD use), and (3) the declining social acceptability of smoking and other ATOD use. In addition, this component includes information and class exercises demonstrating the immediate physiological effects of smoking; presents material concerning media pressures to smoke, drink, or use other drugs; examines techniques used by cigarette and alcoholic beverage advertisers; and teaches skills for resisting alcohol and tobacco advertising as well as peer pressure to smoke, drink, or use other drugs.
- *Personal self-management skills.* This component is designed to (1) improve decisionmaking and problem-solving ability; (2) teach skills for identifying, analyzing, interpreting, and resisting media influences; (3) teach skills for coping with anxiety, anger, and frustration; and (4) provide students with the basic principles of personal behavior change and self-improvement (e.g., goal-setting, self-monitoring, and self-reinforcement).
- *Social skills.* This component is designed to influence several important social skills (e.g., communication, initiating social interactions, conversation, complimenting, skills related to male-female relationships, and verbal and nonverbal assertive skills) and to improve students’ general social competence.

**Figure:**
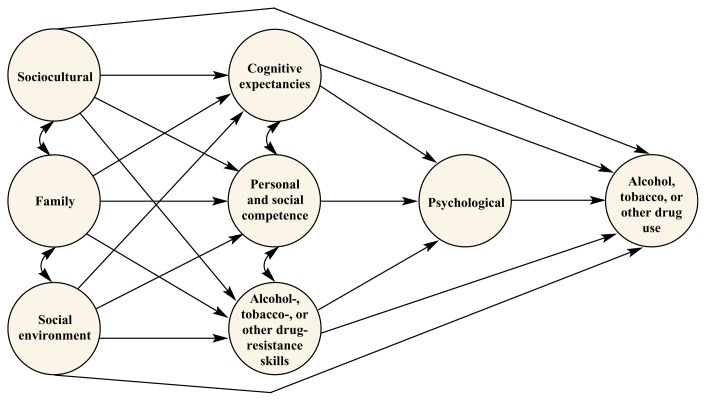
Hypothetical model of initiation of alcohol, tobacco, or other drug use.

### Program Implementation

The LST program consists of 15 class periods (about 45 minutes each) and is intended for middle or junior high school students. In addition to the initial year of intervention in seventh grade, the LST approach continues for 2 additional years. These booster interventions are designed to reinforce the material covered during the first year. There are 10 booster sessions in eighth grade and 5 in ninth grade. For school districts with a middle school, the LST program can be implemented in the sixth, seventh, and eighth grades.

### Program Delivery Methods

The methods used to deliver the content of an effective prevention program may be as important as the content itself. Thus, in developing effective interventions, attention must be given to both the content of the intervention and the delivery methods. A variety of intervention methods have been used in the LST program, including traditional didactic teaching methods, facilitation and group discussion, classroom demonstrations, and cognitive-behavioral skills training. Although lecturing and conventional didactic teaching methods are appropriate for some of the material taught in the LST program, the material can be most effectively taught by facilitating group discussion and focusing on skills training. Because a major emphasis of the LST program is on teaching personal self-management skills, social skills, and skills for resisting social influences to use ATODs, the central role of intervention providers is that of skills trainer or coach. Skills are taught using a combination of the following techniques:

- *Instruction and demonstration.* The first step in the skills training process, instruction and demonstration, consists of explaining how and when to use the skill and then showing students how to perform the skill. The program provider, actors on videotape, or a member of the class can demonstrate the skill.
- *Behavioral rehearsal.* Once the skill has been explained and demonstrated, students practice it using role-playing exercises either at the front of the classroom or in small groups. Role-playing scenarios are clearly described by the provider and are brief (i.e., 1 minute or less each) to ensure that as many students as possible can participate.
- *Feedback.* After the students practice the skills being taught, the provider “critiques” the strengths and weaknesses of each student’s skills “performance.” This information is provided in a gentle and supportive manner so that students understand which aspects of the skill they performed well, which aspects need improvement, and how to improve.
- *Social reinforcement.* The teacher or program provider reinforces or praises each student for one or two positive elements of his or her skills performance. Because the goal of skills training is to improve both the target skills and the self-efficacy of each student, each participant’s improvement is assessed individually.
- *Extended practice.* The final step in the skills training process is extended practice in which students receive behavioral “homework” assignments, such as saying “hello” to one new person each day, practicing a technique for coping with anxiety once per day, and responding assertively in three different situations. In addition to providing opportunities for practice in general, extended practice is intended to facilitate the use of learned skills in situations outside the classroom, promote application to different situations, and encourage students to use the skills as part of their everyday lives. Program providers can further facilitate the skills training process by continuing to provide feedback and reinforcement, as appropriate, outside the classroom.

### Intervention Providers

Several different types of providers have successfully implemented the LST program, including health professionals from outside the school (Botvin et al. 1980, 1994), older peer leaders (Botvin and Eng 1982; Botvin et al. 1984*a*), and regular classroom teachers (Botvin et al. 1983, 1984*a*, 1990). However, the most natural and logical provider for a school-based prevention program is a classroom teacher. Teachers are readily available and generally have more teaching experience and better classroom management skills than other potential intervention providers. Peer leaders (i.e., students who are either the same age or older than the students in training) can assist teachers in implementing the curriculum and serve as positive role models for the kinds of skills and behaviors being taught. Although the LST program has proven to be effective when taught by health professionals, teachers, and peer leaders, the results of one study suggest that peer leaders may produce somewhat stronger prevention effects than do teachers (Botvin et al. 1984*a*).

Provider training can be conducted in 1- or 2-day training workshops or by videotape (Botvin et al. 1990). The purpose of training is to familiarize intervention providers with the program, its rationale, and the results of prior studies as well as give providers an opportunity to learn and practice the skills needed to implement the program successfully (Tortu and Botvin 1989). Peer leader training consists of one half-day workshop in which the prospective peer leaders receive a general orientation to the program as well as an overview of the responsibilities, information, and skills needed to implement the program successfully. In addition, teachers meet with the peer leaders before each session to debrief them on the past session and prepare them for the upcoming one.

## Evidence of Effectiveness

The most serious challenge to the field of ATOD abuse prevention has been in proving that prevention works. Whereas some prevention approaches have been shown to influence knowledge (and, in some cases, attitudes) in a direction consistent with decreased ATOD abuse risk, the gold standard of whether a preventive intervention works is the extent to which it influences ATOD use. Until the late 1970s and early 1980s, little credible evidence existed that prevention efforts worked to reduce ATOD use. Since then, considerable research has been conducted, leading to several promising prevention approaches, including the LST program.

From the 1980s to the present, researchers have conducted a series of studies to test the effectiveness of ATOD abuse prevention approaches based on the LST model. These studies have been conducted in a logical sequence intended to facilitate the development of a prevention approach that is effective with a variety of problem behaviors when implemented by various types of providers and with various populations. Early LST research focused on cigarette smoking and involved predominantly white, middle-class populations. More recent studies, however, have extended this research to other problem behaviors, including the use of alcohol, marijuana and, in one study, other illicit drugs, and have increasingly tested the LST approach among inner-city, minority populations. Furthermore, this research has assessed the long-term durability of the LST prevention model, its impact on hypothesized mediating variables, its implementation fidelity (i.e., the extent to which the intervention is implemented as designed), and methods of improving implementation fidelity. These studies are briefly described in the following sections, along with the key findings, which are expressed by the percent reductions in ATOD use based on a comparison of ATOD rates among students who received the program versus those who did not (i.e., control groups).

### Preventing Tobacco Use

The LST program was initially developed as a smoking prevention program. An early study (Botvin et al. 1980) examined the short-term effectiveness of the LST approach for preventing cigarette smoking among 281 students in the 8th, 9th, and 10th grades. Students at one school received the 10-session prevention program, whereas students at a comparable school served as a control group. Health professionals who were members of the project staff conducted the program. Study results showed a 75-percent reduction in the number of new cigarette smokers at the initial posttest (comparing the posttest smoking rate for the LST group relative to the posttest smoking rate for members of the control group—0.04 vs. 0.16) and a 67-percent reduction in new smoking at the 3-month followup.

In a second study (Botvin and Eng 1982), older peer leaders (11th and 12th graders) served as intervention providers for 7th graders. To emphasize the immediate physical effects of cigarette smoking, a unit was added in which a biofeedback apparatus, such as a heart rate monitor or a tremor tester for assessing hand steadiness, was used in class experiments. This equipment demonstrated the immediate physical effects of smoking on the heart and nervous system among volunteer smokers by showing before and after differences in heart rate and hand steadiness. A methodological improvement (i.e., collecting saliva for analysis before collecting self-report data) was introduced to enhance the validity of self-report smoking data and to provide an objective measure of smoking status. Posttest results indicated that the experimental group included significantly fewer new smokers. These results were corroborated by the results of the saliva thiocyanate (SCN) analysis, which showed a significant increase in smoking among control group students but did not show any increase among experimental group students. In addition, findings indicated a 58-percent reduction in new smoking at the initial posttest and a 56-percent reduction in regular (i.e., weekly) smoking at the 1-year followup. The experimental group also demonstrated significant changes on several hypothesized mediating variables, including smoking knowledge, psychosocial and advertising knowledge, social anxiety, and susceptibility to influence.

A third study (Botvin et al. 1983) examined several important prevention issues, including the efficacy of this approach when implemented by regular teachers, the evaluation of two different implementation schedules, and the efficacy of booster sessions for preserving the initial prevention effects. Seventh-grade students from seven suburban New York schools were randomly assigned to three conditions: (1) a treatment condition in which students were exposed to the prevention program once per week for 15 weeks (group 1); (2) a treatment condition in which students were exposed to the program several times per week for approximately 5 weeks (group 2); and (3) a control condition. As in the previous study, saliva samples were collected to ensure high-quality self-report data.

Significant treatment effects were found at the initial posttest using the measure of monthly smoking. The weekly intervention format (group 1) and the intensive mini-course format (group 2) were equally effective in preventing the onset of new (monthly) smoking. Significant intervention effects for monthly, weekly, and daily smoking were found at the 1-year followup. Groups receiving additional booster sessions had one-half as many regular (i.e., weekly or daily) smokers as groups not receiving booster sessions. Followup conducted one-and-one-half years after the conclusion of the program showed reduced smoking onset rates for monthly, weekly, and daily smoking. These findings provided additional empirical support for the efficacy of the LST prevention program and for its efficacy when conducted by regular classroom teachers. The findings also indicate that LST is effective when implemented according to two different schedules. Perhaps the most important finding of this study, however, is in demonstrating the potential of booster sessions for maintaining and even enhancing the effects of the LST program.

### Preventing Alcohol Use

Several studies have tested the effect of the LST approach on alcohol use frequency, episodes of drunkenness, and heavy drinking. The first of these studies was conducted with 239 seventh graders from two comparable New York City public schools that were randomly assigned to experimental and control conditions (Botvin et al. 1984*b*). The intervention was modified to include material concerning the potential consequences of alcohol use, and where appropriate, skills were taught in relation to situations that might promote alcohol use. Although no effects were evident at the initial posttest, they emerged at the 6-month followup. Compared with students in the control group, significantly fewer (i.e., 54 percent) students in the experimental group reported drinking in the past month, 73 percent fewer reported heavy drinking, and 79 percent fewer reported getting drunk at least once per month.

### Preventing Alcohol and Marijuana Use

A larger study was subsequently conducted to replicate the first study’s findings, to evaluate the LST approach as a strategy for preventing marijuana use in addition to tobacco and alcohol use, and to compare the effectiveness of LST when implemented by older (i.e., 10th- and 11th-grade) peer leaders as opposed to classroom teachers. More than 1,300 seventh-grade students from 10 suburban New York junior high schools participated in the study. The schools were randomly assigned to the following: (1) a teacher-led prevention curriculum, (2) a peer-led prevention curriculum, (3) a teacher-led prevention curriculum and booster sessions, (4) peer-led prevention curriculum and booster sessions, or (5) no prevention curriculum (i.e., a control group).

At the end of the first year, students who participated in the LST program drank significantly less alcohol per drinking occasion and were drunk less often, with the students in the peer-led condition reporting less alcohol use than the students in both the teacher-led and control conditions. In addition, the program reduced experimental marijuana use by 71 percent for students in the peer-led condition and regular (i.e., weekly or daily) marijuana use by 83 percent. The LST program also affected several cognitive, attitudinal, and personality variables consistent with decreased risk of ATOD use (Botvin et al. 1984*b*).

Followup data collected 1 year after the end of the regular intervention showed that depending on the measure used, researchers found 79 to 82 percent fewer smokers in the peer-led booster group compared with the control group and 69 to 78 percent fewer marijuana users in the peer-led booster group compared with the control group. Although no effects were observed overall for the teacher-led group, for students in teacher-led groups in which the teacher taught at least 60 percent of the program (i.e., high implementation fidelity group), there were 44 to 50 percent fewer smokers, 47 percent fewer experimenters with marijuana, and 51 percent fewer drinkers than in the control group (Botvin et al. 1990).

### Long-Term Effectiveness

Most studies that have demonstrated prevention effects have focused on short-term results. To determine the durability of ATOD abuse prevention in general, and the LST approach in particular, we conducted a 6-year randomized trial involving nearly 6,000 students from 56 public schools in New York State. Schools were randomly assigned to prevention and control conditions. Students in the prevention condition received the LST program in the seventh grade, with booster sessions in the eighth and ninth grades. Specially trained classroom teachers taught the prevention program. Prevention effects for tobacco and alcohol use were observed at the end of the intervention (Botvin et al. 1992) as well as at the end of the 12th grade (Botvin et al. 1995*a*). Followup results at the end of the 12th grade indicated that significantly fewer LST students reported smoking cigarettes during the past month and the past week and that significantly fewer students reported heavy smoking (i.e., one pack or more per day). Although researchers found no effects for drinking frequency, significantly fewer prevention students reported getting drunk one or more times per month, compared with students in the control group.

Even stronger prevention effects were found for students in the high implementation fidelity group. Significant prevention effects were found for monthly, weekly, and heavy cigarette smoking as well as for weekly marijuana use. Significant prevention effects were also found for several measures of alcohol use including monthly, weekly, and heavy drinking (i.e., consuming three or more drinks per drinking occasion) as well as for getting drunk one or more times per month.

To assess the effect of the program on more serious levels of ATOD involvement, researchers compared multiple drug use of intervention and control group students. At the end of 12th grade, the researchers found 44 percent fewer LST students than control students who had used tobacco, alcohol, and marijuana one or more times per month and 66 percent fewer LST students who reported using all three substances one or more times per week. The strongest prevention effects were found for the students who received the most complete implementation of the program, including booster sessions.

Finally, although the results showed prevention effects regardless of whether providers were trained at a formal training workshop with periodic feedback and consultation from project staff or through a training videotape, the strongest effects were produced by the teachers who attended annual training workshops and received ongoing support. Both the individual and the school showed prevention effects when used as the unit of analysis.

The results of long-term followup studies can be confounded by differential attrition. For example, when a greater proportion of ATOD users or people at risk for becoming ATOD users in one group cannot be followed up, unbalanced experimental and control groups occur with respect to ATOD use or risk. This phenomenon can undermine the initial (i.e., pretest) equivalence of the treatment and control groups and make it impossible to determine whether any observed followup effects result from intervention or from differential attrition. In this study, attrition rates were equivalent for treatment and control conditions, as were pretest levels of ATOD use for the final analysis sample, supporting the conclusion that the prevention effects resulted from the intervention and not from differential attrition or pretest nonequivalence.

## Effectiveness of LST Among Minority Youth

Researchers have conducted several studies to determine the influence of the LST approach on ATOD use among racial and ethnic minority youth. This work is important not only because it examines the effectiveness of the LST approach among a wider target population, but also because it addresses the gap in ATOD abuse prevention research with minorities.

The most recent LST research with minorities targeted African-Americans and Hispanics. This research initially focused on cigarette smoking and subsequently examined other substances. Although intervention materials and methods were modified, as necessary, throughout development and testing, the underlying prevention strategy was not changed. Modifications related to the reading level at which intervention materials were aimed as well as the inclusion of illustrations or pictures of minority youth and appropriate language, role-playing scenarios, and examples.

### Research With Hispanic Youth

The first study testing the effectiveness of the LST approach among Hispanic youth (Botvin et al. 1989*b*) was conducted with 471 seventh graders (46 percent male) attending eight public schools in the New York metropolitan area. The sample consisted mostly of lower income Hispanic students (74 percent) and included a small percentage of African-American (11 percent) and white (4 percent) students. The schools were randomly assigned to experimental or control conditions. The results indicated significant posttest differences in smoking prevalence between the experimental and control groups after controlling for pretest smoking status, gender, social risk for becoming a smoker, and acculturation. Significant posttest differences also existed between the experimental and control groups on knowledge of the immediate consequences of smoking, smoking prevalence, the social acceptability of smoking, decision-making, normative expectations concerning adult smoking, and normative expectations concerning peer smoking.

A large-scale randomized trial that evaluated the LST approach among a sample predominately composed of urban Hispanic students (Botvin et al. 1992) also found significant effects. More than 3,500 students from 47 public and parochial schools in the greater New York City area participated in the study. We modified intervention materials to increase their relevance to Hispanic youth and to ensure a high degree of cultural sensitivity. Schools were randomly assigned to experimental and control conditions. Using school means (e.g., the average of all the 12th grade students in the followup sample for each school) as the unit of analysis, researchers found significant reductions in cigarette smoking at the end of 7th grade for students who participated in the LST program, relative to the control group. LST students had nearly 30 percent lower monthly prevalence and onset rates compared with students in the control group.

### Research With African-American Youth

Before testing the LST approach with African-American youth, researchers reviewed the intervention materials and methods to determine their cultural appropriateness for this population. Following this review, researchers conducted a small-scale study with nine urban junior high schools in northern New Jersey (Botvin et al. 1989*a*). The pretest involved a total of 608 seventh-grade students, 87 percent of whom were African-American. Schools were randomly assigned to treatment and control conditions within each of the three participating communities. Students in the treatment schools received the LST program, whereas students in the control schools received the smoking education curriculum normally provided.

Results indicated that at posttest, the treatment group had 57 percent fewer smokers than did the control group. Analysis also found significant treatment effects on knowledge of smoking consequences, normative expectations regarding adult smoking prevalence, and normative expectations regarding peer smoking prevalence.

### Tailoring LST to Minority Youth

Although research has demonstrated the generalizability of the LST approach to minority youth, it is often argued that an intervention approach specifically tailored to the target population may have the strongest prevention effects. A recent study tested the relative effectiveness of the LST approach and a prevention approach specifically tailored to African-American and Hispanic youth (Botvin et al. 1994). Both prevention approaches taught students a combination of generic “life skills” and skills specific to resisting offers to use ATODs. The tailored or culturally focused approach, however, was designed to embed the skills-training material in the African-American and Hispanic cultures. Six junior high schools with predominantly minority students were assigned to receive the LST program, receive the culturally focused prevention program, or serve as an information-only control group. The sample was 48 percent African-American, 37 percent Hispanic, 5 percent white, 3 percent Asian, and 8 percent “other.” Students were pretested in the winter and posttested in the spring while in seventh grade.

Results indicated that (1) students in both skills-training conditions expressed lower intentions to drink beer or wine relative to students in the control group and (2) students in the LST condition had lower intentions to drink hard liquor and use illicit drugs. Both skills-training conditions also influenced several mediating variables found to influence the decision not to use ATODs. According to these results, both prevention approaches were equally effective, producing significant reductions in intentions to drink and use illicit drugs and suggesting that a generic ATOD abuse prevention approach with high generalizability may be as effective as a program tailored to individual ethnic populations. As a result, these data provide support for the hypothesis that a single ATOD abuse prevention strategy can be used effectively with multiethnic populations.

Followup data collected 2 years later at the end of the ninth grade found significant effects for both prevention approaches (Botvin et al. 1995*b*). Students in both skills-training conditions drank alcohol less often, became drunk less often, drank less alcohol per drinking occasion, and had lower intentions to use alcohol in the future, relative to students in the control group. These data also showed that the culturally focused intervention produced significantly stronger effects than the generic LST approach. Therefore, the followup study findings suggest that although it may be possible to develop an effective preventive intervention for a relatively broad range of students, tailoring interventions to specific populations can increase effectiveness with inner-city minority populations.

## From Research to Action

For many years the challenges facing prevention researchers and public health professionals have been to identify promising approaches, carefully test them, and provide evidence of their effectiveness. That challenge has largely been met. Much is known at this point about the causes of ATOD abuse and how to prevent it. Although more work is needed to refine existing approaches and develop even more effective ones, the challenge facing us now concerns bridging the gap between research and practice. Recognizing this, efforts are underway to disseminate information about effective ATOD abuse prevention approaches and to move from research to action. During the past few years, national conferences conducted by the National Institute on Drug Abuse, the U.S. Department of Education, and the Justice Department’s Office of Juvenile Justice and Delinquency Prevention have focused on communicating research results indicating that a small group of prevention approaches, such as the LST program, have worked. Speakers at these conferences have exhorted practitioners to use research-based approaches.

One issue that needs to be addressed is cost. Although most prevention programs are inexpensive, schools and communities must be willing to allocate resources to cover the cost of prevention materials and training. The LST program costs range from $5 to $10 per student per year including the cost of materials and training. When several schools in a State or region can coordinate training and the purchase of curriculum materials, the costs can remain closer to $5 per student. Researchers have developed a formal economic analysis of the cost benefit or cost effectiveness of the LST program, but no analysis has been conducted to date. Compared with the expense of treatment or imprisonment, however, which can exceed $30,000 per person per year, the cost of a prevention program like LST is extremely inexpensive.

## Summary, Conclusions, and Future Directions

The etiology of ATOD abuse is complex, involving multiple determinants and numerous developmental pathways. To be effective, prevention approaches should target these determinants as comprehensively as possible. Approaches that focus on ATOD-related knowledge or attitudes have not been found to be effective. Similarly, the use of prevention approaches relying on scare tactics or approaches promoting affective development have failed to influence ATOD use. The most effective prevention approaches have focused on teaching social resistance skills either alone or in combination with general life skills.

LST is currently the most extensively evaluated school-based prevention approach available. More than two decades of research have demonstrated prevention effects with respect to tobacco, alcohol, and marijuana use; multiple substance use; and illicit drug use; as well as hypothesized mediating variables. The magnitude of the reported effects typically has been large, with most studies demonstrating initial reductions of 50 percent or more relative to the control groups. These studies have generally produced reductions in both occasional (i.e., experimental) ATOD use and more serious levels of substance use. Studies of the LST approach have tested its short-term effectiveness, its long-term durability, and the use of different delivery methods as well as the effectiveness of booster sessions, its effectiveness when conducted by different program providers, and its effectiveness with different populations. These studies have ranged from small-scale pilot studies involving two schools and a few hundred adolescents to large-scale, multisite, randomized field trials involving more than 50 schools and several thousand adolescents.

Although considerable progress has been made in the past decade, further research is needed. To this end, additional research is underway in order to better understand the mechanisms through which the LST approach prevents ATOD use; to understand the LST program’s effectiveness with different populations (particularly minority youth); and to determine the program’s generalizability to other empirically and theoretically related behaviors, such as violence. One obvious limitation of this type of prevention approach is that it addresses a limited set of etiological factors within school settings. More research is therefore needed either to extend this approach to other intervention modalities (e.g., approaches targeting the family or community) or to integrate it within a more comprehensive intervention strategy targeting a broader array of etiological factors.
